# The role of routine post-natal abdominal ultrasound for newborns in a resource-poor setting: a longitudinal study

**DOI:** 10.1186/1471-2431-11-64

**Published:** 2011-07-12

**Authors:** Atinuke M Agunloye, Adejumoke I Ayede, Samuel I Omokhodion

**Affiliations:** 1 Department of Radiology, College of Medicine and University College Hospital, University of Ibadan, Ibadan, Nigeria; 2 Department of Pediatrics, College of Medicine and University College Hospital, University of Ibadan, Ibadan, Nigeria

## Abstract

**Background-:**

Neonatal abdominal ultrasound is usually performed in Nigeria to investigate neonatal symptoms rather than as a follow up to evaluate fetal abnormalities which were detected on prenatal ultrasound. The role of routine obstetric ultrasonography in the monitoring of pregnancy and identification of fetal malformations has partly contributed to lowering of fetal mortality rates. In Nigeria which has a high maternal and fetal mortality rate, many pregnant women do not have ante-natal care and not infrequently, women also deliver their babies at home and only bring the newborns to the clinics for immunization. Even when performed, most routine obstetric scans are not targeted towards the detection of fetal abnormalities.

The aim of the present study is to evaluate the benefit of routinely performing abdominal scans on newborns with a view to detecting possible abnormalities which may have been missed ante-natally.

**Methods-:**

This was a longitudinal study of 202 consecutive, apparently normal newborns. Routine clinical examination and abdominal ultrasound scans were performed on the babies by their mother's bedside, before discharge. Neonates with abnormal initial scans had follow-up scans.

**Results-:**

There were 108 males and 94 females. There were 12 (5.9%) abnormal scans seen in five male and seven female neonates. Eleven of the twelve abnormalities were in the kidneys, six on the left and five on the right. Three of the four major renal anomalies- absent kidney, ectopic/pelvic kidney and two cases of severe hydronephrosis were however on the left side. There was one suprarenal abnormality on the right suspected to be a possible infected adrenal haemorrage. Nine of the abnormal cases reported for follow- up and of these, two cases had persistent severe abnormalities.

**Conclusions-:**

This study demonstrated a 5.9% incidence of genito urinary anomalies on routine neonatal abdominal ultrasound in this small population. Routine obstetric USS is very useful but inadequate availability of skilled personnel and cost implications create great challenges in poor resource settings like Nigeria. However, awareness should be created so that parents who can afford such investigations can make informed decisions.

## Background

Neonatal abdominal ultrasound is usually performed as a follow up to further evaluate fetal abnormalities which were detected on prenatal ultrasound or in the course of investigating neonatal symptoms.

The role of the prenatal ultrasound has evolved in its specificity (93-99%) and sensitivity (14-85%) for identification of fetal malformations over the last five decades and has partly contributed to the lowering of fetal mortality rates [1-9]. More abnormalities are seen by the third trimester and a single early scan may miss some fetal anomalies. This implies that some abnormalities may still be missed ante-natally.

In the South-Western part of Nigeria and perhaps all of the country, most neonatal abdominal scans are performed to investigate neonatal symptoms. The role of ultrasound in this case is usually to confirm or exclude congenital or acquired, inflammatory or neoplastic lesions of abdominal organs. Since most routine obstetric scans in Nigeria are not targeted towards the detection of fetal abnormalities and there are no national guidelines for these studies, babies are frequently born with gross abnormalities not previously detected on single or multiple pre-natal scans. There are many cases in which mothers do not have ante-natal care and present in labour. Not infrequently women also deliver their babies at home and only bring the newborns to the clinics for immunization.

The aim of the present study is to evaluate the benefit of routinely performing abdominal scans on newborns with a view to detecting possible congenital abnormalities which may have been missed ante-natally.

This is a longitudinal study of 202 apparently normal newborns that had abdominal ultrasound scans performed on the lying-in ward before discharge. Neonates with abnormal initial scans had follow-up scans.

## Methods

This was a longitudinal study. Ethical approval for the study was obtained from the Oyo State Research Ethical Review Committee (OYSRERC, Reference number AD 13/262/183). Written consent was obtained from the parents/caregiver of the neonates. Translation of the consent form was done to the local language and applied when needed.

This study was carried out at the University College Hospital (UCH), Ibadan, over a period of one year between May 2009 and May 2010. There were a total of 1,800 deliveries over the study period. Of these, abdominal Ultrasound scan (USS) was carried out on 202 consecutive apparently normal newborn babies whose parents/care givers gave written consent.

Routine clinical examination was performed on all the newborns by the pediatrician to exclude any obvious congenital abnormality. The USS was performed before the mother and child were discharged, usually within the first 5 days of life.

The scans were done at the mother's bedside using a SONOSITE portable USS machine with Doppler facilities. All scans were performed by AMA, the consultant radiologist. A curvilinear transducer with frequency range of 5-7.5MHz was used on the neonates following application of a water-based, non-allergenic ultrasound gel. Multiple views of the abdomen were acquired to visualize all the abdominal organs. If neonatal hydronephrosis was present the Society for Fetal Urology, America (SFU) grading was used (table 1). Neonates with abnormal USS findings had follow-up scans.

**Table 1:** Society for Fetal Urology (SFU) grading system of congenital hydronephrosis based on longitudinal ultrasound scan of the kidneys

Grade	Central renal complex	Renal parenchymal thickness
0	Intact	Normal
1	Urine in pelvis barely splits sinus	Normal
2	Evident splitting of pelvis and major calyces	Normal
3	Wide splitting of pelvis, major & minor calyces	Normal
4	Further splitting of pelvis, major & minor calyces	Reduced

## Results

Of the 202 neonates studied, 108 were males while 94 were females with a male to female ratio of 1.1:1. Table 2 shows the gender distribution of the study subjects and age at which the USS were performed.

**Table 2:** Distribution of the sex and age at which the USS was performed in 202 neonates

Age in Days	Male (%)	Female (%)	Total (%)
1	23 (21.3)	27 (28.7)	50 (24.8)
2	26 (24.1)	23 (24.5)	49 (24.3)
3	22 (20.4)	18 (19.1)	40 (19.8)
4	13 (12.0)	16 (17.0)	29 (14.4)
5	16 (14.8)	2 (2.1)	18 (8.9)
6	6 (5.6)	7 (7.4)	13 (6.4)
7	2 (1.9)	1 (1.1)	3 (1.5)
**Total**	**108 (53.5)**	**94(46.5)**	**202 (100.0)**

**Figure 1:**
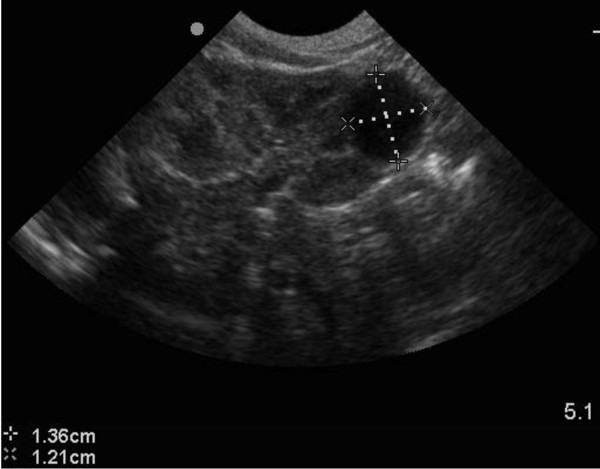
**USS of left kidney showing a cortical cyst in inferior pole**.

**Figure 2:**
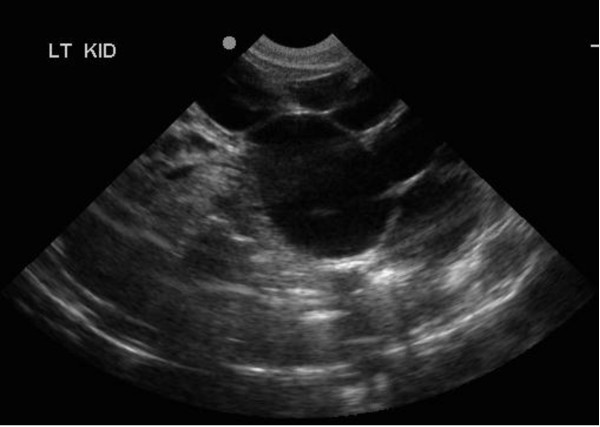
**USS of left kidney on 4^th ^day of life showing dilated renal pelvis and calyces, renal parenchyma is thinned**.

**Figure 3:**
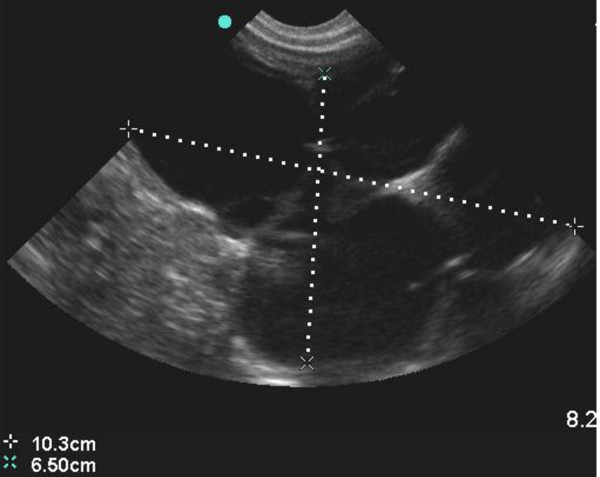
**Follow-up scan of left kidney in figure 2 showing worsened calyceal dilatation**.

**Figure 4:**
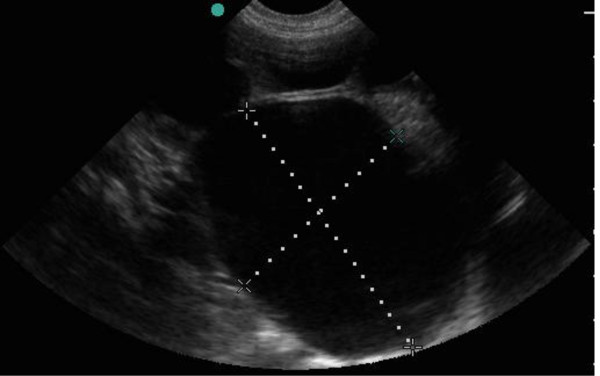
**Follow-up scan of left kidney in figure 2 showing worsened dilatation of the renal pelvis**.

There were 12 (5.9%) abnormal scans seen in five male and seven female neonates. Eleven of the twelve abnormalities were in the kidneys, six on the left and five on the right. Three of the four major renal anomalies- absent kidney, ectopic/pelvic kidney and two cases of severe uretero-pelvic junction obstructions (UPJO) were however on the left side. There was one suprarenal abnormality on the right suspected to be a possible haemorrage (table 3 and figures 1, 2, 3 and 4). The baby with the suprarenal mass was admitted due to suspected infected suprarenal haemorrage and managed with antibiotics. The mass gradually resolved and was not visualized at the 6^th ^month follow-up scan.

**Table 3:** shows the abnormal USS findings at initial and follow up scans

Serial no	Age in days	sex	Initial USS findings	*Follow-up USS findings*
1.	3	F	Absent left kidney	***Follow up at 18 months-*** *Absent Left Kidney.* *Normal Right Kidney (7.9 × 3.4 cm)*
2.	4	F	Mild dilated right renal pelvis. SFU grade 1	***Follow up at 18 months-*** *Right kidney-Extra-renal pelvis, normal calyces. RK- 6.6 × 2.6 cm; LK- 6.5 × 2.5 cm*
3	2	M	Pelvic (Ectopic) right kidney	***Follow up at 18 months-*** *Rt pelvic kidney- 5.9 × 2.7 cm; LK-5.9 × 2.5 cm*
4	1	M	Malrotated left kidney	***Follow up at 18 months****- normal* *RK- 5.6 × 2.6 cm; LK-5.7 × 2.4 cm*
5	4	F	Duplex right collecting system	***Lost to follow up***
6	2	F	Right Suprarenal/Adrenal Mass-Hypoechoic suggestive of haemorrage (3.4 × 1.8 cm) Fever, jaundice on day 2	***4 weeks****- Echogenic, smaller adrenal mass (1.4 × 0.9 cm)* ***6 months****- Total resolution of mass*
7	2	F	Left calyceal cyst- upper pole	***Lost to follow up***
8	1	F	Left cortical cyst- inferior pole (1.4 × 1.2 cm)- fig 1	***4 weeks****- cyst = 1.1 × 1.1 cm* ***6 months****- normal USS. No cyst*
9	5	M	Mild dilated right renal pelvis. SFU grade 1	***6 months****- Normal USS*
10	1	M	Mild dilated right renal pelvis. SFU grade 1(HIV positive mother)	***Lost to follow up***
11	2	F	Left Hydronephrosis SFU grade 3	***6 weeks ****-Left hydronephrosis-SFU grade 3 (LK- 5.5 × 3 cm; RK- 4.9 × 2.2 cm)* ***4 1/2 months- ****Left hydronephrosis SFU grade 3 (LK- 5.8 × 2.4 cm; RK- 5.7 × 2.4 cm)*
12	4	M	Left Hydronephrosis SFU grade 4.- figure 2 LK = 8.9 × 5.3 cm; RK = 4.5 × 1.7 cm.	***4 months****- SFU grade 4* *LK = 10.3 × 6.5 cm; RK = 5.5 × 2.5 cm -*figure 3 and figure 4

**USS**- Ultrasound; **LK**- Left kidney; **RK**- Right Kidney; **SFU**- Society for Fetal Urology Grade.

Nine abnormal cases reported for follow- up while three cases were lost to follow-up despite repeated phone calls and attempt to locate their houses using the parent's documented phone numbers and addresses. The abnormal renal findings with SFU grade 3 and 4 persisted while the cases with SFU grade 1 and the cysts resolved. The cases with persistent abnormalities were referred to the Paediatric surgeon/Urologist for further management

## Discussion

There are conflicting reports on the optimal time to perform a postnatal abdominal scan for urological abnormalities after birth [1]. Proponents of delaying scan till 72 hours after birth suggest that earlier scans may be misleading due to relative oliguria in the first 72 hours of life which may lead to underestimation of the degree of hydronephrosis [1,10]. However, other studies have not corroborated this claim [11,12] and it is technically more convenient to perform the scans before the mother and neonate are discharged home, usually within 48 hours after birth, as was done in this study. The default cases who failed to keep their follow-up visits also suggest that pre-discharge scans are more desirable in the study area.

On prenatal ultrasound, the most frequently seen fetal abnormalities are those of the urinary system. Of these, hydronephrosis is the commonest, seen in about 50% of such cases [13], and it occurs commonly in males [14]. Fetal USS evaluation of the urinary system is possible from the 15^th ^week of gestation [15] but USS at about 32 weeks gestation is the best time for detecting these abnormalities as an earlier scan in the same fetus may have been normal [16].

For cases of hydronephrosis not diagnosed in-utero, the role of post-natal abdominal ultrasound will be to determine the cases due to obstruction which can lead to renal damage and therefore require surgical intervention or long term follow-up of renal function [1]. If hydronephrosis is seen in the fetal kidneys, the SFU grading system or the renal pelvis diameter (RPD) measurement is used to determine cases that need post-natal follow up with USS, micturating cysto-urethrogram (MCUG) or diuretic renogram. MCUG is done to rule out associated vesico-ureteric reflux (VUR) seen in 20%- 33% of patients and posterior uretheral valves (PUV) which may co-exist in patients with bilateral hydronephrosis [17,18]. Diuretic renography is useful to evaluate the degree of obstruction and determine differential renal function [17,19]. There are no specific guidelines for these cases in our institution but the two cases with persistent hydronephrosis were referred for MCUG which is available in our centre.

Up to 60% of ante-natally detected cases of hydronephrosis resolve spontaneously [10,14,17,20,21] and the threshold limit for spontaneous resolution of fetal or neonatal hydronephrosis has been put at RPD between 5 mm-20 mm and SFU grade 1 to 2 by several authors [16,17,22-27]. This corroborates with findings in this study where persistent hydronephrosis was seen only in the cases with SFU grades 3 and 4 up to four months of age. It is however generally agreed that conservative management options should initially be considered for most patients. If postnatal USS is normal after 4 to 6 weeks of age, further USS follow- up is unnecessary [28].

Uretero-pelvic junction obstruction (UPJO) is the commonest cause of hydronephrosis due to upper urinary tract obstruction in children [12,29] and is seen in 1 in 1000-1500 births. There are intrinsic or extrinsic causes and males are twice to thrice as affected as females [12,30-32]. It is bilateral in 10-40% of affected patients with the left side being twice as affected as the right [12,29,32]. Classic USS findings are dilated calyces and renal pelvis with normal ureter [33,34] and this was seen in the two cases with SFU grades 3 and 4 hydronephrosis who require long term follow-up. About 25% of cases will have clinical and functional deterioration requiring surgical repair but there is increasing trend towards conservative management [12,35]. The decision for surgical intervention depends on the function of the affected kidney and the status of the other kidney at initial assessment [29,36]. Since 13-42% of patients with UPJO have associated vesico-ureteric reflux, MCUG is advised in all patients with this condition as was done in the above cases [12]. The two cases with suspected UPJO had normal findings on MCUG with no evidence of VUR or PUV noted. As diuretic renography is not available in our centre, the patients are being followed up with serial ultrasound twice a year to monitor degree of hydronephrosis and renal parenchymal thinning and if these are progressive, surgical intervention will be considered.

The ectopic kidney is one which lies outside the normal renal fossa (at the level of the 1^st ^to 3^rd ^Lumbar vertebrae) and is usually in the pelvis but may rarely be in the posterior thorax. The ectopic kidney may cross over to the contralateral side where it may fuse with the second kidney (crossed renal ectopia) [37]. The incidence of ectopic kidney is about 1 in 5000 from screening studies [38] and 1 in 1000 from post-mortem studies [39]. Ectopic kidneys are associated with increased incidence of other urological abnormalities especially VUR and are also prone to increased risk of trauma [37,40,41]. In a study by Lusch *et al *[42,22], 6% of children with pelvic kidneys were symptomatic with recurrent urinary tract infection (UTI), abdominal pain, hypertension and hydronephrosis. Regular USS follow up once or twice yearly was suggested for such symptomatic cases. The mother of the neonate with pelvic kidney in this study was counseled on these possible complications and recommended management.

Absence of the kidney (Renal agenesis) may occur but the bilateral form is rare, commoner in males and is incompatible with life. Prenatal USS would show oligohydramnios and a persistently undistended fetal urinary bladder [37]. Unilateral renal agenesis is however fairly commonly seen in about 1 in 500 births. It is associated with absent ipsilateral ureter, hemitrigone and renal artery and may also be malrotated or ectopic in location [37]. The adrenal gland will be present and may mimic the kidney on USS. In such patients, the anatomical and functional status of the second kidney needs to be confirmed by USS, intravenous urography and nuclear scintigraphy. There is usually compensatory hypertrophy of the second kidney with good prognosis if it is functioning normally but any damage by infection, calculus disease, trauma or reflux may be lethal [37]. However, further tests could not be performed on the patient with suspected left renal agenesis as she was lost to follow-up.

Malrotation of the kidney is the commonest but least significant of renal abnormalities. It refers to an abnormal relationship between the renal pelvis and renal tissue. The condition may be isolated when it can be unilateral or bilateral; or be associated with other renal anomalies like ectopic kidney. UPJO is a common complication [37,38]. Duplex collecting system of the kidney is the commonest anomaly of the upper collecting system and ureter (ureteropelvic duplication) resulting from premature division of the ureteral bud or simultaneous development of two ureteral buds [37,43]. The pattern of abnormality ranges from bifid renal pelvis (incomplete type) to complete duplication of the ureter. The former is twice as common as the latter and unilateral cases are five times as common as bilateral cases for either type. The clinical significance of this condition is dependent on the ureteric insertion, whether normal or ectopic. Duplication of the renal collecting system as seen in this study is diagnosed on ultrasound when the central echo complex is divided into two with an interposed column of renal parenchyma [43].

Caliceal diverticular cysts may be congenital or acquired and they communicate with the calyces or renal pelvis. It may be associated with hydronephrosis and diagnosed ante-natally [44]. They may be asymptomatic but about 50% are associated with stones [45] but this was not seen in the case above.

The incidence of incidental adrenal masses on imaging has been put at between 0.6 to 1.3% and is higher with abdominal CT scan [46]^.^Adrenal tumors and adrenal haemorrhage can be diagnosed with USS and it has been reported that prenatal USS diagnosis of neuroblastoma results in a higher survival rate as it is identified at an early stage [47]. Adrenal haemorrhage results from multiple patho-physiological factors. It is seen in about 1.9 per 1000 live births [43,48] and it is the commonest cause of adrenal mass in the neonate, usually presenting between day 2 and day 7 of life [43,49]. It is more commonly seen in neonates than in children or adults because the neonatal gland is about two times larger and therefore prone to hypotension and asphyxia [50]. The neonate with suspected adrenal haemorrhage in this study also had jaundice which is a known association [43,51,52]. Other known associations include a palpable flank mass, anaemia and hypovolemic shock but it could also be asymptomatic. Serial imaging with ultrasound until complete resolution is advised for these cases as was done for this patient in whom complete resolution was seen at six months. USS is the examination of choice in neonates with suspected adrenal hematoma. Initial USS typically shows a complex, echogenic mass with inferior displacement of the kidney if the bleed is large. Over time, the mass becomes smaller, cystic and echolucent over a period of weeks. It may also subsequently develop calcifications [44]. The USS appearance is however variable as seen in this case where the initial finding was a hypoechoic mass on second day of life, which subsequently became smaller and echogenic at six weeks and disappeared by six months.

The limitations of this study include the small sample size and the cases lost to follow up, more babies need to be scanned to be able to make pronouncements on the incidences of documented abnormal cases in the study area. Another limitation is that all ultrasound scans were performed by a single operator (AMA). This was because only one portable ultrasound machine was available to perform bedside scans which ensured the cooperation of mothers and performance of scans before discharge from hospital. However, AMA is an experienced sonologist with over twelve years experience as a consultant sonologist.

## Conclusions

This study has been able to demonstrate a 5.9% incidence of various types of genito urinary anomalies in this small population. Routine pre- and post- natal USS has been known to be very useful in early identification and prompt intervention of congenital genito urinary abnormalities in the fetus/newborn. However, inadequate availability of skilled personnel and cost implication of such investigations create great challenges in poor resource settings like Nigeria. Even though the cost benefit of early diagnosis and prompt treatment of significant renal abnormalities is high, recommending routine neonatal abdominal/renal USS will most likely be hampered by persistent low socioeconomic status of most Nigerians. Public awareness on the possibility of detecting these cases early with resultant better prognosis should however be created such that parents who can afford such investigations can make informed decisions.

## Competing interests

The authors declare that they have no competing interests.

## Authors' contributions

AMA and AIA conceived, designed the study and acquired the data. All authors (AMA, AIA and SO) were involved in the analysis and interpretation of data; drafting and revision of manuscript. All authors read and approved the final manuscript.

## Pre-publication history

The pre-publication history for this paper can be accessed here:

http://www.biomedcentral.com/1471-2431/11/64/prepub
